# Construction and Application of a Korean Reference Panel for Imputing Classical Alleles and Amino Acids of Human Leukocyte Antigen Genes

**DOI:** 10.1371/journal.pone.0112546

**Published:** 2014-11-14

**Authors:** Kwangwoo Kim, So-Young Bang, Hye-Soon Lee, Sang-Cheol Bae

**Affiliations:** 1 Department of Rheumatology, Hanyang University Hospital for Rheumatic Diseases, Seoul, Republic of Korea; 2 Division of Rheumatology, Immunology and Allergy, Brigham and Women's Hospital, Harvard Medical School, Boston, Massachusetts, United States of America; 3 Division of Genetics, Brigham and Women's Hospital, Harvard Medical School, Boston, Massachusetts, United States of America; 4 Program in Medical and Population Genetics, Broad Institute, Cambridge, Massachusetts, United States of America; Centro di Riferimento Oncologico, IRCCS National Cancer Institute, Italy

## Abstract

Genetic variations of human leukocyte antigen (HLA) genes within the major histocompatibility complex (MHC) locus are strongly associated with disease susceptibility and prognosis for many diseases, including many autoimmune diseases. In this study, we developed a Korean HLA reference panel for imputing classical alleles and amino acid residues of several HLA genes. An HLA reference panel has potential for use in identifying and fine-mapping disease associations with the MHC locus in East Asian populations, including Koreans. A total of 413 unrelated Korean subjects were analyzed for single nucleotide polymorphisms (SNPs) at the MHC locus and six HLA genes, including *HLA-A*, *-B*, *-C*, *-DRB1*, *-DPB1*, and *-DQB1*. The HLA reference panel was constructed by phasing the 5,858 MHC SNPs, 233 classical HLA alleles, and 1,387 amino acid residue markers from 1,025 amino acid positions as binary variables. The imputation accuracy of the HLA reference panel was assessed by measuring concordance rates between imputed and genotyped alleles of the HLA genes from a subset of the study subjects and East Asian HapMap individuals. Average concordance rates were 95.6% and 91.1% at 2-digit and 4-digit allele resolutions, respectively. The imputation accuracy was minimally affected by SNP density of a test dataset for imputation. In conclusion, the Korean HLA reference panel we developed was highly suitable for imputing HLA alleles and amino acids from MHC SNPs in East Asians, including Koreans.

## Introduction

There is a well-characterized high degree of genetic variability in human leukocyte antigen (HLA) genes located at the major histocompatibility complex (MHC) locus on chromosome 6 [1]. Although diverse genetic variations and heterozygosity of HLA molecules allow the immune system to defend against a wide range of foreign molecules and pathogens [2], various classical alleles of HLA genes have been found to be associated with susceptibility to or prognosis of multiple inflammatory disorders and other complex traits [3], [4].

Despite the clinical importance of the locus, the decision to genotype HLA alleles for a large number of subjects is limited by the high cost of genotyping and low throughput genotyping methods. Currently, it is possible to infer HLA alleles from SNPs in the MHC locus using several HLA imputation methods, such as SNP2HLA and HLA*IMP [5], [6]. SNP2HLA has the advantage of being able to identify the most likely causal variants in complex diseases like HIV infection, rheumatoid arthritis, multiple sclerosis, and follicular lymphoma, because this method can impute amino acid residues in HLA genes as well as classical HLA alleles [7]-[11]. SNP2HLA uses a specialized HLA reference panel that encodes SNPs within the MHC region along with HLA classical alleles and amino acid residues [5]. However, this method has not been used in HLA association studies in non-European populations because the HLA reference panel was generated from European genetic information. To overcome this limitation, the first Asian HLA reference panel was very recently constructed mostly from Southeast Asian subjects [11], [12].

In this study, we developed an HLA reference panel for East Asians, including Koreans, for use with SNP2HLA.

## Materials and Methods

The genetic data of 413 unrelated Korean subjects were included in our new HLA reference panel. The subjects were examined for their SNPs by Illumina's HumanOmniExpress array at SNPgenetics Inc. (Seoul, South Korea). We applied general quality control criteria for minor allele frequency (MAF; MAF >0.5%), Hardy-Weinberg equilibrium (HWE; p value of HWE >5×10^−7^), call rate per SNP (>98%), call rate per individual (>98%), heterozygosity rate, population stratification, sex consistency between subject-reported and genetic sex, and cryptic relatedness (duplicate and cryptic 1st degree relatives). SNPs within the MHC from 25 Mb to 33 Mb on chromosome 6 (in hg18) were extracted to construct the HLA reference panel.

Classical HLA alleles of *HLA-A*, *-B*, *-C*, *-DRB1*, *-DPB1* and *-DQB1* were genotyped using Roche's GS 454 sequencing at the Institute for Immunology and Infectious Diseases (Murdoch WA, Australia). The institutional calling algorithms for HLA alleles were accredited by the American Society for Histocompatibility and Immunogenetics (ASHI). To examine sequencing and calling accuracy for the HLA allele, we independently genotyped the classical allele *HLA-DRB1* at 4-digit resolution for all subjects by a conventional sequencing method using a WAKFlow HLA typing kit (Hiroshima, Japan) at Labgenomics Clinical Laboratories (Seongnam, Korea).

All genotyped data of the MHC SNPs, HLA alleles, and HLA amino acid residues were phased by Beagle 3.0.4 in SNP2HLA with some modifications. HLA amino acid residues were assigned from the amino acid sequence information of each 4-digit HLA-allele.

Imputation accuracy of the reference panel was examined by comparing the imputed and genotyped results of classical HLA alleles. To this end, test subjects (n  = 83) and reference subjects (n = 330) were randomly selected from the 413 Korean subjects 100 times at a 1∶4 ratio. Using each pair of test and reference subsets, we imputed HLA alleles from MHC SNPs in the test subjects by using SNP2HLA and the reference panel from the matched reference subjects. We then calculated 100 concordance rates between imputed and genotyped classical alleles of each HLA gene and obtained an average concordance rate for each gene.

In addition, we imputed HLA alleles from the HapMap3 r2 SNP dataset of East Asian HapMap population [Han Chinese from Beijing, China (CHB) and Japanese from Tokyo, Japan (JPT)] using the reference panel, including all Korean 413 reference samples. To compare the imputed HLA alleles with actual HLA alleles, the HLA data of CHB + JPT HapMap population for *HLA-A*, *-B*, *-C*, *-DRB1* and *-DQB1* was obtained from two independent studies [13], [14]. Individuals with discordant results between the two studies were excluded from this analysis. *HLA-A*, -B, -C, and *-DRB1* were genotyped in the two studies. Of 89 CHB + JPT subjects in the HapMap3 r2 dataset, 61 subjects were exactly matched for all 4-digit alleles of the HLA genes in the two studies.

To evaluate the imputation accuracy of an ethnicity-mismatched HLA reference panel, a European reference panel (constructed from data collected by the Type 1 Diabetes Genetics Consortium; T1DGC) was used to impute for the East Asian HapMap3 r2 dataset [5], [13]. To ensure panel size consistency, we created subsets of the European reference panel by randomly selecting 413 individuals and independently repeated this process 100 times. Imputation accuracy was evaluated using the mean of the 100 independent concordance rates for each HLA gene.

The study was approved by the Institutional Review Board of Hanyang University (IRB file No. HYUH 2001-06-001), and written consent was obtained from study participants.

## Results and Discussion

A total of 5,858 polymorphic MHC SNPs in the 413 study subjects passed our quality control criteria. The 4-digit alleles of the 6 HLA genes (*HLA-A*, *-B*, *-C*, *-DRB1*, *-DPB1* and *-DQB1*) typed using NGS methods were assigned to 233 HLA alleles, which results in 1,025 polymorphic amino acid positions in the study subjects (**Table 1**). The frequencies of HLA alleles in the Korean subjects are shown in **Table S1 in File S1**. Given our sample size (826 haplotypes), we expected to detect at least 4.1 times the HLA alleles with frequency ≥0.5% in the general Korean population.

**Table 1:** Number of classical HLA alleles and polymorphic amino acid positions in the HLA reference panel.

Gene	HLA allele markers (2-digit/4-digit)	Amino acid positions
*HLA-A*	11/23	144
*HLA-B*	25/44	139
*HLA-C*	11/25	216
*HLA-DRB1*	13/34	208
*HLA-DPB1*	11/13	192
*HLA-DQB1*	5/18	126
Total	76/157	1,025

The reliability of the calling algorithm in the NGS-based genotyping method was evaluated by comparing *HLA-DRB1* alleles with conventionally genotyped results for all of the study subjects. A concordance rate of 99.52% was observed at 4-digit resolution of *HLA-DRB1* alleles.

The HLA reference panel encoded all information about each of the MHC SNPs, HLA-alleles, and amino acid residues as binary codes, such as absence/presence or allele1/allele2, as previously described [5]. We evaluated the newly constructed reference panel by assessing imputation accuracy using the MHC SNPs in a test subset among the subjects (n = 83; 20%) and the reference panel from the reference subset comprised of all remaining subjects (n = 330; 80%). The test subset and the reference subset were randomly selected 100 times and each pair of test and reference subsets was used in an imputation analysis by SNP2HLA. When we surveyed all imputed data and compared the data with actual genotype data, the average genotype concordance rates were 95.4% and 90.8% at 2-digit and 4-digit allele resolution, respectively. For each HLA gene, concordance ranged from 91.3% (for *HLA-B*) to 97.9% (for *HLA-A*) at 2-digit allele resolution and from 85.9% (for *HLA-B*) to 95.0% (for *HLA-DPB1*) at 4-digit allele resolution (**Table 2**).

**Table 2:** Concordance rates between imputed and genotyped alleles.

Reference panel	Test dataset	Allelic resolution	HLA gene						
			A	B	C	DRB1	DPB1	DQB1	Total
Korean* (n = 330)	Korean* (n = 83)	2-digit	0.979	0.913	0.965	0.931	0.970	0.970	0.954
		4-digit	0.908	0.859	0.928	0.868	0.950	0.937	0.908
Korean (n = 413)	CHB + JPT (n = 61)	2-digit	0.934	0.934	0.992	0.959	NA	0.967	0.957
		4-digit	0.910	0.893	0.984	0.893	NA	0.893	0.915
European** (n = 413)	Korean (n = 413)	2-digit	0.920	0.510	0.770	0.622	0.886	0.740	0.712
		4-digit	0.766	0.455	0.687	0.400	0.863	0.650	0.592
European** (n = 413)	CHB + JPT (n = 61)	2-digit	0.911	0.283	0.668	0.754	NA	0.794	0.682
		4-digit	0.829	0.233	0.602	0.591	NA	0.659	0.583

* Reference panel and test datasets were randomly assigned 100 times from all 413 Korean subjects and the mean concordance rate was calculated by 100 independent imputations using each of the 100 reference panel and test dataset pairs.

** For consistency of panel size between Korean and European panels, the mean concordance rate was calculated by 100 independent imputations using 100 different subsets (n = 413) of the European reference panel.

In addition, we imputed the HLA alleles from MHC SNPs of East Asian HapMap individuals (CHB + JPT) using the Korean reference panel (n = 413) to assess imputation accuracy from an independent Asian dataset. The average concordance rates between genotyped and imputed alleles were 95.7% (93.4 - 99.2%) at the 2-digit allele resolution and 91.5% (89.3 - 98.4%) at the 4-digit allele resolution, which are similar to those in the cross-tested Korean subjects. Pearson's correlation coefficients between actual and imputed dosage of classical HLA alleles were calculated to evaluate the imputation accuracy at the allele level. The new reference panel showed a high correlation between imputed and actual dosage (average correlation coefficient r = 0.887 for alleles with frequency ≥0.01 and r = 0.735 for alleles with frequency <0.01; **Figure S1 in File S1**).

In contrast, when we used a European reference panel for imputing HLA alleles of the East Asian HapMap subjects, imputation accuracy from the ethnicity-mismatched reference panel was much worse than that from the Korean reference panel, especially for *HLA-B*, *-C*, *-DRB1* and *-DQB1* (**Table 2**). The observed ethnicity effect in imputation has been consistently reported in previous studies [5], [11], [12], [15].

**Figure 1:**
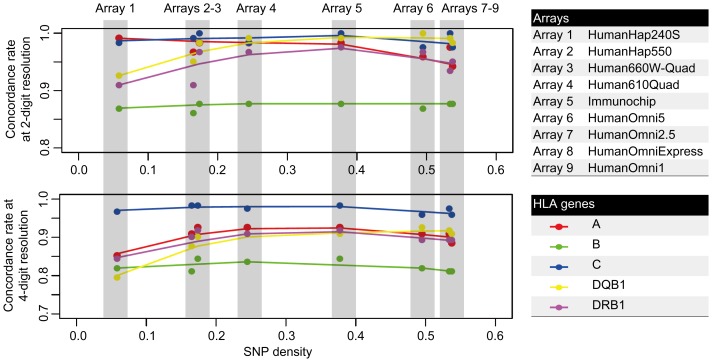
Concordance rates using various reference panels. SNPs present in 9 different commercial arrays were extracted for imputing the HLA alleles from the HapMap3 r2 dataset of East Asian (CHB + JPT) individuals. Each subset was imputed for *HLA-A*, *-B*, *-C*, *-DQB1* and *-DRB1* using the Korean reference panel. Concordance rates (y-axis) were plotted against the proportion of reference-panel SNPs that were present in each subset (x-axis).

We further investigated the effect of SNP density in test datasets on imputation accuracy in the Korean HLA reference panel. From the CHB + JPT HapMap dataset, we extracted nine sets of SNPs that were present in each of nine commercial genome-wide SNP arrays instead of using all SNPs in the HapMap3 r2 datasets. Using a low-density test dataset dramatically decreased the number of SNPs that were present in both the reference panel and the array-based test dataset. However, SNP density had little effect on imputation accuracy (**Figure 1**). Accuracy was slightly decreased only by use of very low-density genotyping arrays, which are no longer commercially available.

To illustrate the advantage of the new Korean reference panel in an association study, we applied the reference panel to our previous SNP-based case-control association study [16] on rheumatoid arthritis (RA) that shows the strongest association at *HLA-DRB1* within the extended MHC region [11]. After imputing HLA variants from the SNP-based dataset [16], we compared disease effect sizes of the imputed *HLA-DRB1* alleles with those of the genotyped *HLA-DRB1* alleles that were obtained in our previous HLA-allele-based case-control association study [17]. We note that there is a some portion of overlapping subjects (n = 2,355) between the SNP-based (n = 9,299) and HLA-based datasets (n = 3,034) [16], [17], and that we already fully dissected and reported HLA-RA associations down to the amino acid level in our another recent paper [11].

**Figure 2:**
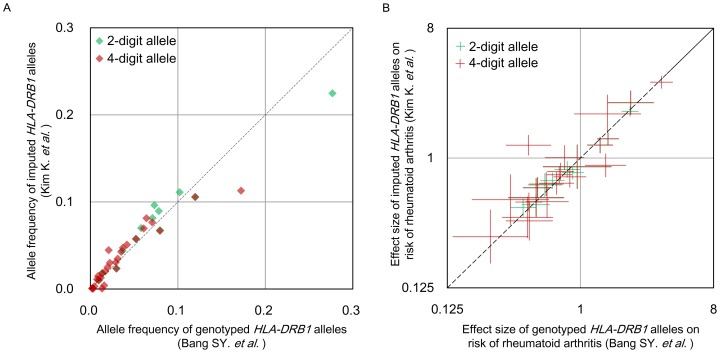
Frequencies and disease effect sizes of imputed and genotyped *HLA-DRB1* alleles. We revisited our previous rheumatoid arthritis association studies using either typed SNPs [16] or HLA alleles [17]. After imputing HLA variants from the SNP-based dataset, (A) frequencies and (B) disease effect sizes of the imputed classical alleles of *HLA-DRB1* were compared with those of genotyped classical alleles in the HLA-based dataset.

We found highly correlated allele frequencies between imputed and genotyped *HLA-DRB1* alleles (correlation coefficient r = 0.976 at 2-digit resolution and r = 0.932 at 4-digit resolution; **Figure 2A**), although a large portion of subjects was not examined in the both studies. The effect sizes of each HLA allele were also highly concordant between the two datasets (r = 0.984 at 2-digit resolution and r = 0.938 at 4-digit resolution; **Figure 2B**), which demonstrating reliability, validity and usefulness of the Korean reference panel in MHC-disease association studies.

## Conclusion

The Korean HLA reference panel constructed in our study was found to be highly applicable and suitable for various genome-wide array data from East Asians, including Han Chinese, Japanese, and Korean populations. The Korean reference panel is publicly available from **File S2** and https://sites.google om/site/scbaehanyang/hla_panel/.

## Supporting Information

File S1 **Tables S1; Figures S1.**
(PDF)

File S2 **Korean HLA reference panel.**
(ZIP)
