# Supplementary material for: Construction and Application of a Korean Reference Panel for Imputing Classical Alleles and Amino Acids of Human Leukocyte Antigen Genes
Source: PLoS One. 2014 Nov 14;9(11):e112546. doi: 10.1371/journal.pone.0112546 (PMC4232350; doi:10.1371/journal.pone.0112546)

Supplementary information (Kim K *et al*)

Table S1. Frequency of each allele of *HLA-A*, *-B*, *-C*, *-DPB1*, *-DQB1*, and *-DRB1* in 413 Korean subjects

A	Freq.	B	Freq.	C	Freq.	DPB1	Freq.	DQB1	Freq.	DRB1	Freq.
*01:01	0.0242	*07:02	0.0424	*01:02	0.1646	*02:01	0.2785	*02:01	0.0242	*01:01	0.0654
*02:01	0.1416	*07:05	0.0073	*01:03	0.0048	*02:02	0.0424	*02:02	0.0642	*01:60	0.0012
*02:03	0.0073	*08:01	0.0024	*02:02	0.0061	*03:01	0.0375	*03:01	0.1295	*03:01	0.0230
*02:06	0.0981	*13:01	0.0133	*03:02	0.0775	*04:01	0.0763	*03:02	0.1029	*04:01	0.0012
*02:07	0.0327	*13:02	0.0400	*03:03	0.1053	*04:02	0.0896	*03:03	0.1126	*04:03	0.0315
*02:10	0.0061	*14:01	0.0061	*03:04	0.1005	*05:01	0.3644	*03:29	0.0012	*04:04	0.0073
*03:01	0.0133	*15:01	0.0860	*03:13	0.0012	*09:01	0.0170	*03:33	0.0012	*04:05	0.0860
*03:02	0.0012	*15:02	0.0036	*03:43	0.0012	*13:01	0.0496	*04:01	0.0860	*04:06	0.0424
*11:01	0.1090	*15:07	0.0109	*04:01	0.0630	*14:01	0.0145	*04:02	0.0291	*04:07	0.0048
*11:02	0.0024	*15:11	0.0170	*05:01	0.0121	*15:01	0.0036	*05:01	0.0860	*04:08	0.0036
*24:02	0.2312	*15:17	0.0012	*06:02	0.0642	*17:01	0.0230	*05:02	0.0303	*04:10	0.0109
*24:08	0.0012	*15:18	0.0157	*07:01	0.0266	*29:01	0.0024	*05:03	0.0424	*04:51	0.0012
*24:20	0.0024	*15:27	0.0024	*07:02	0.0860	*38:01	0.0012	*05:54	0.0048	*07:01	0.0714
*26:01	0.0375	*15:38	0.0024	*07:04	0.0073			*06:01	0.0835	*08:02	0.0230
*26:02	0.0206	*18:01	0.0012	*08:01	0.0872			*06:02	0.0835	*08:03	0.0775
*26:03	0.0036	*27:04	0.0024	*08:02	0.0061			*06:03	0.0206	*09:01	0.0848
*29:01	0.0073	*27:05	0.0218	*08:03	0.0133			*06:04	0.0521	*10:01	0.0170
*30:01	0.0363	*35:01	0.0811	*08:06	0.0012			*06:09	0.0460	*11:01	0.0472
*30:04	0.0073	*35:03	0.0048	*12:02	0.0157					*11:04	0.0036
*31:01	0.0460	*37:01	0.0206	*12:03	0.0048					*12:01	0.0496
*31:11	0.0012	*38:02	0.0121	*12:05	0.0012					*12:02	0.0303
*32:01	0.0048	*39:01	0.0073	*14:02	0.0666					*13:01	0.0206
*33:03	0.1646	*40:01	0.0400	*14:03	0.0557					*13:02	0.0956
		*40:02	0.0375	*15:02	0.0206					*13:67	0.0012
		*40:03	0.0061	*15:05	0.0073					*14:01	0.0339
		*40:06	0.0327							*14:03	0.0109
		*44:02	0.0121							*14:05	0.0291
		*44:03	0.0799							*14:06	0.0061
		*46:01	0.0581							*14:07	0.0024
		*47:01	0.0012							*15:01	0.0848
		*48:01	0.0521							*15:02	0.0218
		*51:01	0.0823							*16:01	0.0012
		*51:02	0.0085							*16:02	0.0085
		*51:06	0.0012							*16:09	0.0012
		*52:01	0.0145								
		*54:01	0.0484								
		*55:01	0.0024								
		*55:02	0.0085								
		*56:01	0.0036								
		*56:05	0.0012								
		*57:01	0.0036								
		*58:01	0.0714								
		*59:01	0.0266								
		*67:01	0.0061								

Figure S1. Dosage correlation between imputed and genotyped alleles

Imputed dosages (0 to 2) of 2-digit alleles (red) and 4-digit alleles (green) of *HLA-A*, *-B*, *-C*, *-DQB1*, and *-DRB1* in CHB+JPT HapMap individuals were compared with the actual dosage (0, 1 or 2). The correlation coefficient between the imputed and actual dosages of each allele was plotted according to its allelic frequency.

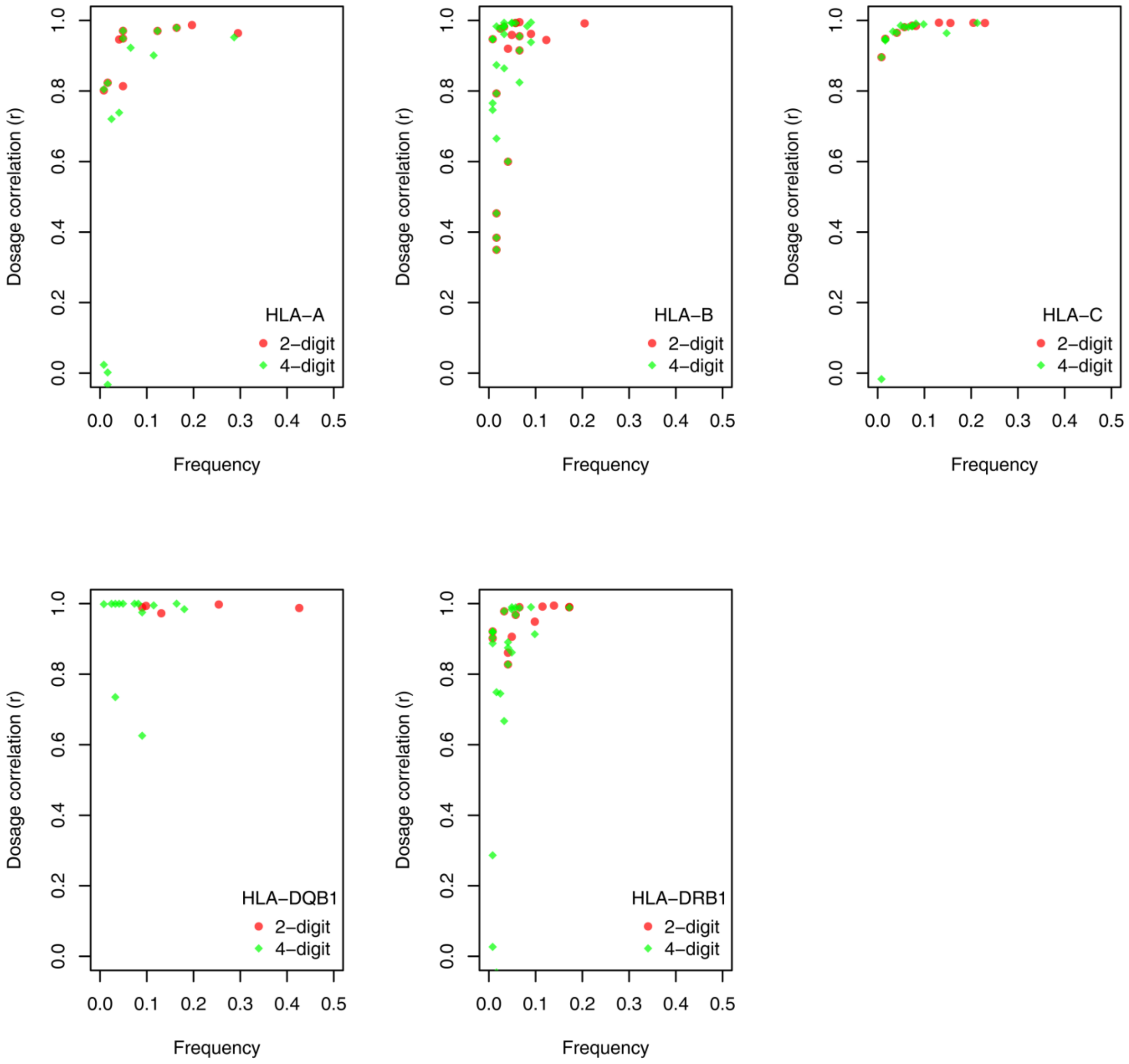

Supplement: File S1 — Tables S1; Figures S1. (PDF) [file pone.0112546.s001.pdf]
